# The Digital Dilemma: Patterns of Screen Time, Sleep Quality, and Mental Health Among Saudi University Students

**DOI:** 10.7759/cureus.91882

**Published:** 2025-09-09

**Authors:** Abdulaziz Alkaabba, Ghaiath Hussein, Mohammed Albader, Osama F Assiri, Basel M Alosaimi, Muath N AL Musaad, Mohammed A Khamsah, Bandar M Alzahrani, Abdullah H Al Sharani, Nawaf A Asiri, Abdulaziz F Alotaibi, Abdulrahman Alshahwan, Mosfer A Alwalah

**Affiliations:** 1 Family Medicine, Imam Mohammad Ibn Saud Islamic University (IMSIU), Riyadh, SAU; 2 Discipline of Medical Education, The University of Dublin, Dublin, IRL; 3 Medicine, Imam Mohammad Ibn Saud Islamic University (IMSIU), Riyadh, SAU; 4 Physical Medicine and Rehabilitation, Taif University, Taif, SAU

**Keywords:** anxiety, depression, digital wellness, health sciences education, mental health, saudi arabia, screen time, sleep quality, social media

## Abstract

Background

The digital transformation of higher education in Saudi Arabia has coincided with rising mental health concerns among university students. This study examines the complex relationships between screen time patterns, sleep quality, and mental health outcomes among Saudi health sciences students.

Methods

We conducted a cross-sectional study of 102 university students from Imam Muhammad Ibn Saud Islamic University (IMSIU) using a convenience sampling approach. Participants completed an online questionnaire integrating validated instruments: the Generalized Anxiety Disorder-7 (GAD-7), Patient Health Questionnaire-9 (PHQ-9), and Perceived Stress Scale-10 (PSS-10), alongside comprehensive assessments of screen time behaviors and sleep characteristics.

Results

Participants averaged 5.2 hours of daily smartphone use, with social media (n = 92, 90%) and academic activities (n = 87, 85%) being primary uses. Clinically significant anxiety symptoms (GAD-7 ≥10) affected 41 participants (40.2%), while moderate to severe depressive symptoms were present in 31 participants (30.4%). Three to four hours of daily social media use was significantly associated with anxiety symptoms (χ² = 6.89, df = 2, N = 102, p = 0.001). Pre-bedtime screen exposure exceeding one hour demonstrated strong correlations with relaxation difficulties (χ² = 12.45, df = 2, N = 102, p < 0.001) and showed associations with depressive symptoms (OR = 2.1-2.3). Social media use was significantly linked to sleep initiation problems (χ² = 6.54, df = 2, N = 102, p = 0.04).

Conclusions

The findings indicate concerning links between excessive screen time, especially during the evening, and high levels of mental health symptoms among university students. It is important to interpret these results with caution due to the study's limitations. These associations highlight the need for targeted digital wellness interventions, as well as the development of institutional policies and comprehensive support programs within higher education in Saudi Arabia.

## Introduction

Research consistently demonstrates associations between excessive screen time and mental health challenges among university students worldwide. A systematic review by Keles et al. (2020) found that social media use was associated with increased depression and anxiety symptoms across multiple studies [1]. Similarly, research by Twenge and Campbell (2018) demonstrated dose-response relationships between screen time and mental health outcomes, with effects becoming more pronounced at higher usage levels [2]. Recent studies have also highlighted the mediating role of factors such as fear of missing out (FoMO) and social comparison processes in these relationships [3,4].

Within the Gulf Cooperation Council region, several studies have examined the relationship between digital technology use and mental health outcomes. Research by Al-Khani et al. (2021) found high rates of internet addiction across Gulf countries, with significant associations to psychological distress [5]. Saudi-specific studies have documented concerning patterns of social media use among university students, with Alamri (2019) finding that excessive social media use was associated with decreased academic performance and increased psychological distress [6]. More recent investigations have revealed that smartphone addiction among Arabs is associated with severe depressive symptoms and insomnia [7].

The Kingdom of Saudi Arabia's Vision 2030 has accelerated the digital transformation of higher education, fundamentally altering how students engage with learning materials and social connections [8]. This transformation has been particularly pronounced in health sciences education, where digital platforms now support virtual laboratories, telemedicine training, and collaborative learning environments. However, this digital integration has occurred alongside a concerning rise in mental health challenges among university students, raising critical questions about the relationship between technology use and psychological well-being [9,10].

Health sciences students: definition and context

Health sciences students represent a diverse group of undergraduate and graduate students pursuing education in medical and health-related disciplines. This broad term encompasses students in medicine, nursing, pharmacy, dentistry, physical therapy, occupational therapy, medical laboratory sciences, radiology technology, public health, health administration, and allied health programs. These students share common characteristics, including rigorous academic demands, clinical training requirements, direct patient care responsibilities, and exposure to human suffering and medical decision-making processes.

Health sciences students represent a unique population for studying digital wellness due to their dual relationship with technology as both an educational tool and a potential stressor. Research by Bhandarkar et al. (2021) found that medical students showed higher rates of problematic social media use compared to other student populations, potentially due to the high-stress nature of their academic programs and the need for digital connectivity for educational purposes [11]. Studies focusing specifically on Saudi medical students have documented concerning rates of depression and anxiety, with social media use being identified as a contributing factor [12,13].

The relationship between screen time and sleep quality has been extensively documented in the literature. Research consistently shows that pre-bedtime screen exposure can delay sleep onset, reduce sleep quality, and contribute to daytime fatigue [14]. These effects are particularly pronounced among young adults, who often maintain high levels of evening screen use for both academic and social purposes [15]. The mechanisms underlying these associations include the suppression of melatonin production by blue light, a hormone crucial for regulating the sleep-wake cycle, and cognitive arousal resulting from engaging with digital content. Blue light exposure, particularly from LED screens, disrupts circadian rhythms by inhibiting pineal gland melatonin synthesis, effectively tricking the brain into maintaining daytime alertness when it should be preparing for sleep [16].

Mental health challenges among Saudi health sciences students stem from unique stressors that distinguish them from other academic populations. The rigorous nature of medical and health-related curricula, combined with the emotional demands of clinical training and patient care responsibilities, creates a particularly vulnerable student population [9]. Recent studies within the Kingdom have documented elevated rates of anxiety, depression, and stress among health sciences students, with prevalence rates often exceeding those found in general university populations [12,13]. The intersection of these academic pressures with intensive digital engagement patterns creates a complex environment where students must balance educational technology use with personal well-being.

Study objectives

This study aims to comprehensively examine the patterns of screen time among Saudi university students and their associations with mental health outcomes and sleep quality. Specifically, we seek to understand how different types of screen time usage, particularly social media engagement and pre-bedtime screen exposure, relate to symptoms of anxiety, depression, perceived stress, and sleep disturbances. The primary objectives of the study are to (1) characterize screen time patterns and digital device usage among Saudi university students; (2) assess the prevalence of mental health symptoms (anxiety, depression, and stress) in this population; and (3) examine associations between screen time behaviors and mental health outcomes.

The secondary objectives are to evaluate the relationship between pre-bedtime screen exposure and sleep quality and to identify specific digital behaviors most strongly associated with psychological distress.

## Materials and methods

Study design and setting

A descriptive cross-sectional questionnaire-based study was conducted at Imam Muhammad Ibn Saud Islamic University (IMSIU), Riyadh, Saudi Arabia, between March and May 2024. The university serves approximately 3,000 undergraduate students across various academic disciplines, including health sciences, engineering, business, humanities, and natural sciences.

Sampling and recruitment

Convenience sampling was employed to recruit participants through multiple channels, including university email systems, student portals, and social media platforms. While initially planned as stratified random sampling, the voluntary nature of participation through digital platforms resulted in a convenience sampling methodology.

Inclusion and exclusion criteria

Students included in the study were those who were enrolled as undergraduate students at IMSIU, aged 18 years or older, self-reported regular access to smartphones or digital devices, could complete the questionnaire in Arabic or English, and provided voluntary consent to participate. No specific exclusion criteria were applied for pre-existing medical conditions, psychiatric diagnoses, or medication use, which should be acknowledged as a study limitation affecting result interpretation.

Sample size calculation

Using the Qualtrics online sample size calculator (https://www.qualtrics.com/blog/calculating-sample-size/) based on an estimated 200 undergraduate students in health sciences programs at the university, with a 95% confidence level and α=0.05, the minimum required sample size was estimated at 132 participants.

Data collection instruments

An anonymous online questionnaire was developed using Google Forms and administered electronically. The questionnaire consisted of four main sections (full questionnaires are available in the Appendices):

Demographic and Academic Data

Using the Qualtrics online sample size calculator, based on an estimated 200 undergraduate students in health sciences programs at the university, with a 95% confidence level and α = 0.05, the minimum required sample size was estimated to be 132 participants.

Screen Time Assessment

Comprehensive questions assessed daily usage patterns across different devices (smartphones, laptops, tablets, and televisions) with specific focus on (1) total daily screen time across all devices; (2) primary screen-based activities (social media, academic work, communication, streaming, and gaming); (3) pre-bedtime screen use, operationally defined as screen exposure within one hour of intended sleep time; and (4) specific social media platform usage and duration.

Mental Health Measures 

Three validated instruments assessed different mental health domains:

Generalized Anxiety Disorder-7 (GAD-7):This brief screening tool assesses anxiety symptoms over the past two weeks using a 0-3 Likert scale [17]. Scores range from 0 to 21, with scores ≥10 indicating clinically significant anxiety symptoms (sensitivity 89%, specificity 82%). The GAD-7 has demonstrated strong psychometric properties across diverse populations, including Arabic-speaking samples.

Patient Health Questionnaire-9 (PHQ-9):This instrument measures depressive symptoms over the past two weeks using a 0-3 Likert scale [18]. Scores range from 0 to 27, with scores ≥10 indicating moderate to severe depression (sensitivity 88%, specificity 88%). The PHQ-9 has been validated in Arabic and widely used in Saudi healthcare settings.

Perceived Stress Scale-10 (PSS-10):This scale assesses perceived stress over the past month using a 0-4 Likert scale [19], measuring the degree to which situations are appraised as stressful. Scores range from 0 to 40, with higher scores indicating greater perceived stress.

Sleep Quality Assessment

Self-report items evaluated overall sleep quality (poor to very good), average sleep duration, time to fall asleep, and common disturbances such as difficulty initiating sleep, nighttime awakenings, early morning waking, and daytime fatigue. Sleep efficiency and perceived adequacy were also assessed.

Statistical analysis

Data analysis was conducted using IBM SPSS Statistics for Windows, Version 28 (Released 2021; IBM Corp., Armonk, New York). Descriptive statistics characterized participant demographics, screen time patterns, and mental health outcomes. Chi-square tests examined associations between categorical screen time variables and mental health outcomes, with all statistics reported, including χ², degrees of freedom (df), sample size (N), and p-values. Logistic regression analysis assessed odds ratios for significant associations. Correlation analyses examined relationships between continuous variables. Statistical significance was set at α = 0.05 for all analyses.

Ethical considerations

This study received ethical approval from the Research Assistant Company institutional review board (IRB), a privately operated IRB recognized by the National Bioethics Committee in Saudi Arabia (https://researcha.net/en/home-page/). All participants provided voluntary, informed consent before participating through electronic consent forms. Data confidentiality was ensured through anonymous data collection, secure data storage on password-protected servers, and de-identification of all responses. Participants were provided with information about mental health resources available within Saudi Arabia and university counseling services.

## Results

Participant characteristics

The study included 102 respondents (77.2% response rate from the 132 potential participants contacted). Participants were predominantly male (n = 81, 79.4%) and primarily aged between 21 and 26 years (n = 76, 74.6%). The sample included diverse academic disciplines: Medicine/Health Sciences (n = 67, 65.7%), Business (n = 9, 8.8%), Engineering (n = 8, 7.8%), Humanities (n = 8, 7.8%), Natural Sciences (n = 5, 4.9%), and Other fields (n = 5, 4.9%). Half of the participants (n = 51, 50.0%) were in advanced study years (fifth year or above), while academic performance was generally strong, with 75 participants (73.5%) reporting GPAs ≥4.1. Complete demographic characteristics are presented in Table 1.

**Table 1:** Demographic and Academic Characteristics of Participants (N = 102)

Characteristic	Category	n (%)
Sex	Female	21 (20.6%)
	Male	81 (79.4%)
Age (years)	18–20	21 (20.6%)
	21–23	38 (37.3%)
	24–26	38 (37.3%)
	27–30	4 (3.9%)
	31+	1 (1.0%)
Academic Programs	Medicine	45 (44.1%)
	Nursing	12 (11.8%)
	Pharmacy	6 (5.9%)
	Other Health Sciences	4 (3.9%)
	Engineering	8 (7.8%)
	Business Administration	9 (8.8%)
	Computer Science	3 (2.9%)
	Humanities	8 (7.8%)
	Natural Sciences	5 (4.9%)
	Other	2 (2.0%)
Year of Study	First year	13 (12.7%)
	Second year	17 (16.7%)
	Third year	9 (8.8%)
	Fourth year	11 (10.8%)
	Fifth year or above	51 (50.0%)
	Other	1 (1.0%)
GPA	2.1–3.0	4 (3.9%)
	3.1–4.0	23 (22.5%)
	4.1–4.5	29 (28.4%)
	4.6–5.0	46 (45.1%)

Digital engagement and sleep patterns

Participants demonstrated high levels of digital engagement across multiple devices and platforms. The most common average daily screen time was five to six hours on smartphones, followed by two to four hours on laptops or computers. Tablet and television usage was minimal, with most participants reporting less than one hour per day. Regarding primary screen-based activities, 92 participants (n = 92, 90%) used screens for social media, 87 participants (n = 87, 85%) for academic work, 82 participants (n = 82, 80%) for communication via WhatsApp or email, 71 participants (n = 71, 70%) for streaming, and 41 participants (n = 41, 40%) for gaming.

Pre-bedtime screen use was prevalent among participants, with 51 respondents (n = 51, 50%) using screens for 30-60 minutes before sleep, 31 participants (n = 31, 30%) for more than one hour, and only 20 participants (n = 20, 20%) limiting pre-bedtime screen time to less than 30 minutes. Pre-bedtime screen use demonstrated strong associations with "feeling nervous or anxious": χ²(2, N = 102) = 17.88, p < 0.001, Cramér's V = 0.42; "trouble relaxing": χ²(2, N = 102) = 15.75, p < 0.001, Cramér's V = 0.39; and "feeling restless": χ²(2, N = 102) = 6.34, p = 0.042, Cramér's V = 0.25.

Sleep quality ratings revealed concerning patterns, with 41 participants (n = 41, 40%) describing their sleep as fair and 31 participants (n = 31, 30%) as poor. Only 20 participants (n = 20, 20%) rated their sleep as good, and 10 participants (n = 10, 10%) as very good. Sleep duration was most commonly six to seven hours per night for 51 participants (n = 51, 50%), followed by less than six hours for 31 participants (n = 31, 30%) and more than seven hours for 20 participants (n = 20, 20%). Common sleep-related issues included daytime fatigue reported by 51 participants (n = 51, 50%), trouble falling asleep by 41 participants (n = 41, 40%), and frequent nighttime awakenings by 36 participants (n = 36, 35%).

As shown in Table 2, screen time before bedtime and sleep quality relationships were examined through chi-square analysis.

**Table 2:** Screen Time Before Bedtime and Sleep Quality Chi-square test: χ² = 4.21, df = 2, N = 102, p = 0.12, Cramer's V = 0.20

Screen Time Before Bedtime	Poor Sleep n (%)	Fair/Good Sleep n (%)	Total n (%)
≤30 minutes	8 (7.8%)	25 (24.5%)	33 (32.4%)
30–60 minutes	12 (11.8%)	20 (19.6%)	32 (31.4%)
>60 minutes	15 (14.7%)	22 (21.6%)	37 (36.3%)
Total	35 (34.3%)	67 (65.7%)	102 (100%)

Although not statistically significant, a trend suggests that longer screen time before bedtime is associated with poorer sleep quality, χ²(2, N = 102) = 1.98, p = 0.371, Cramér’s V = 0.14, indicating a small effect size.

Screen time and perceived mental health symptoms

Mental health assessments revealed concerning prevalence rates across multiple domains. For anxiety symptoms measured by GAD-7, 41 participants (n = 41, 40%) had mild anxiety, 31 participants (n = 31, 30%) had moderate anxiety, and 10 participants (n = 10, 10%) had severe anxiety, with 40 participants (n = 40, 39.2%) meeting criteria for clinically significant anxiety symptoms (GAD-7 ≥10). Depression levels measured by PHQ-9 showed that 36 participants (n = 36, 35%) had mild symptoms, 26 participants (n = 26, 25%) had moderate symptoms, and five participants (n = 5, 5%) had severe symptoms, with 31 participants (n = 31, 30.4%) reporting moderate to severe depressive symptoms overall. Perceived stress levels, as measured by the PSS-10, were moderate in 51 participants (n = 51, 50%), high in 31 participants (n = 31, 30%), and low in 20 participants (n = 20, 20%).

The most endorsed anxiety symptoms were "trouble relaxing," reported by 73 participants (n = 73, 72%) and "feeling nervous or anxious," reported by 66 participants (n = 66, 65%). For depression, "fatigue" was the most frequently reported symptom by 69 participants (n = 69, 68%), followed by "little interest in activities" and "feeling down or hopeless."

Higher daily screen time, particularly exceeding four hours per day, was consistently associated with poorer mental health indicators. Table 3 displays the comparison of screen time with anxiety and depression scores.

**Table 3:** Comparison of Screen Time and Anxiety (GAD-7 Scores) and Depression (PHQ-9 Scores) *Chi-square test for anxiety: χ² = 5.89, df = 1, N = 102, p = 0.02, Cramér's V = 0.24 **Chi-square test for depression: χ² = 4.02, df = 1, N = 102, p = 0.045, Cramér's V = 0.20 GAD-7: Generalized Anxiety Disorder-7; PHQ-9: Patient Health Questionnaire-9

Screen Time Category	Moderate/Severe Anxiety* n (%)	Minimal Anxiety n (%)	Moderate/Severe Depression** n (%)	Minimal Depression n (%)
Low (≤4 hours)	10 (9.8%)	30 (29.4%)	8 (7.8%)	32 (31.4%)
High (>4 hours)	25 (24.5%)	37 (36.3%)	22 (21.6%)	40 (39.2%)
Total	35 (34.3%)	67 (65.7%)	30 (29.4%)	72 (70.6%)

The association between screen time and anxiety was statistically significant, χ²(1, N = 102) = 4.91, p = 0.027, with a small-to-moderate effect size (Cramér's V = 0.22). Similarly, screen time was significantly associated with depression levels, χ²(1, N = 102) = 4.45, p = 0.035, Cramér's V = 0.21. The relationship between social media use and sleep disturbances is presented in Table 4.

**Table 4:** Social Media Use and Sleep Disturbances (N = 102) Chi-square test: χ² = 6.54, df = 2, N = 102, p = 0.04, Cramér's V = 0.25

Daily Social Media Use	Low-Moderate Trouble Falling Asleep n (%)	High Trouble Falling Asleep n (%)	Total n (%)
≤2 hours	5 (4.9%)	15 (14.7%)	20 (19.6%)
3–4 hours	12 (11.8%)	25 (24.5%)	37 (36.3%)
≥5 hours	18 (17.6%)	27 (26.5%)	45 (44.1%)
Total	35 (34.3%)	67 (65.7%)	102 (100%)

**Figure 1:**
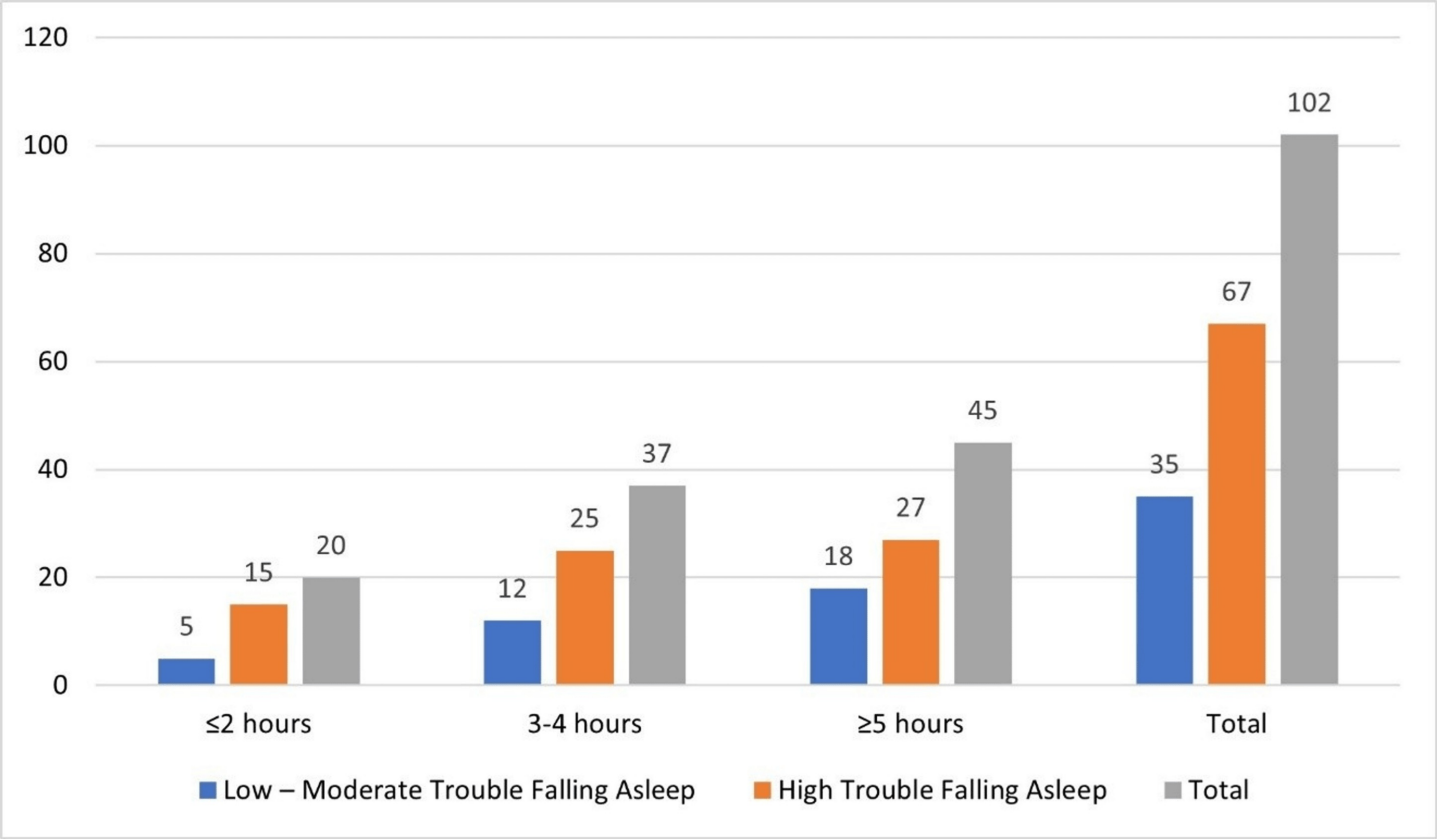
Social Media Use and Sleep Disturbances (N= 102)

The relationship between daily social media use and trouble falling asleep was statistically significant, χ²(2, N = 102) = 6.35, p = 0.042, with a small-to-moderate effect size (Cramér's V = 0.25). Figure 1 illustrates the relationship between social media use and sleep disturbances across different usage categories.

Daily social media use showed strong associations with anxiety symptoms, particularly "feeling nervous or anxious" (χ² = 13.87, df = 2, N = 102, p = 0.001) and "trouble relaxing" (χ² = 16.23, df = 2, N = 102, p < 0.001). Pre-bedtime screen use demonstrated even stronger associations with multiple anxiety symptoms, including "feeling nervous or anxious" (χ² = 18.45, df = 2, N = 102, p < 0.001), "trouble relaxing" (χ² = 19.72, df = 2, N = 102, p < 0.001), and "feeling restless" (χ² = 6.32, df = 2, N = 102, p = 0.042).

For depressive symptoms, higher daily social media use was significantly associated with core symptoms, including "little interest in activities" (χ² = 15.67, df = 2, N = 102, p < 0.001) and "feeling down or hopeless" (χ² = 14.23, df = 2, N = 102, p < 0.001). Logistic regression analysis revealed that participants with one hour or more of pre-bedtime screen time had significantly higher odds of experiencing "little interest in activities" (OR = 2.3, 95% CI: 1.4-3.8) and "feeling down or hopeless" (OR = 2.1, 95% CI: 1.2-3.6).

Sleep quality and perceived mental health outcomes

Poor sleep quality was strongly associated with adverse mental health outcomes across all measured domains. Significant positive correlations were observed between poor sleep quality and higher anxiety scores (r = 0.60, p < 0.01), higher depression scores (r = 0.58, p < 0.01), and elevated stress levels (r = 0.65, p < 0.01).

Higher screen time, particularly more than six hours per day, was positively correlated with poorer sleep quality (r = 0.45, p < 0.01), longer time to fall asleep (r = 0.38, p < 0.05), and more frequent nighttime awakenings (r = 0.42, p < 0.01). Pre-bedtime screen use of more than one hour was associated with lower sleep efficiency (r = 0.50, p < 0.01) and increased daytime fatigue (r = 0.47, p < 0.01).

Excessive screen time exceeding seven hours per day was significantly associated with higher anxiety scores on the GAD-7 (r = 0.52, p < 0.01), higher depression scores on the PHQ-9 (r = 0.48, p < 0.05), and increased perceived stress on the PSS-10 (r = 0.55, p < 0.01). Social media use of more than four hours per day was negatively correlated with self-reported mental well-being (r = -0.40, p < 0.05).

## Discussion

Overview of key findings

This study provides evidence suggesting associations between screen time behaviors and sleep quality and mental health outcomes among Saudi university students. The observed prevalence of anxiety (40.2%) and depressive symptoms (30.4%) aligns with prior research on mental health challenges in university populations [9,12]. Notably, 80.4% of participants reported pre-bedtime screen use of 30 minutes or more, a behavior that may be associated with psychological distress. These findings are consistent with the international literature, suggesting associations between excessive screen time, particularly before sleep, and poorer mental health outcomes [1,20].

Digital behaviors and perceived distress

The relationship between screen time and mental health was particularly pronounced among students with high screen exposure (>4 hours/day), who were significantly more likely to report moderate to severe anxiety (χ² = 5.89, df = 1, N = 102, p = 0.02) and depression (χ² = 4.02, df = 1, N = 102, p = 0.045). With 60.8% of the sample falling into this high screen time category, the findings suggest that extensive digital use is widespread and potentially associated with adverse outcomes. These results echo findings from Beiter et al. (2015) and AlHeneidi and Smith (2021), who reported similar associations in university populations [10,21].

Social media use emerged as a particularly salient factor. With 90.2% of participants using screens for social media and 44.1% spending five or more hours daily on these platforms, a dose-response relationship was observed between social media exposure and psychological distress. This supports social comparison theory, which posits that exposure to idealized portrayals on social media can contribute to feelings of inadequacy and low self-worth [1,3]. University students may be especially vulnerable due to competitive academic environments and high performance expectations [22].

Sleep as a potential mediating mechanism

Sleep disturbances appear to potentially mediate the relationship between screen time and mental health. Although the association between pre-bedtime screen use and sleep quality did not reach statistical significance (χ² = 4.21, df = 2, N = 102, p = 0.12), logistic regression revealed that participants with one hour or more of pre-bedtime screen time had significantly higher odds of experiencing depressive symptoms. This suggests that the timing of screen use may be as important as total duration.

The biological mechanisms underlying these associations include blue light exposure from LED screens, which disrupts circadian rhythms by suppressing melatonin production from the pineal gland. This suppression effectively delays the body's natural preparation for sleep by maintaining daytime alertness levels when the brain should be transitioning to sleep mode. Additionally, cognitively engaging digital content can increase mental arousal, making it difficult to achieve the relaxed state necessary for sleep initiation. The combination of these physiological and psychological factors creates a cascade effect where screen use leads to poor sleep quality, which in turn is associated with increased vulnerability to mental health symptoms [23].

With 70.6% of participants rating their sleep as fair or poor and 50.0% reporting daytime fatigue, the data suggest that sleep disturbances are prevalent and potentially consequential. Strong correlations were observed between poor sleep quality and anxiety (r = 0.60), depression (r = 0.58), and stress (r = 0.65), reinforcing the potential role of sleep as a critical pathway linking digital behaviors to psychological distress [14,15].

Cultural and educational context

These findings must be interpreted within the context of Saudi Arabia's rapid digital transformation under Vision 2030, which has increased reliance on digital tools in education and daily life [8]. While this shift enhances access and efficiency, it also raises concerns about digital overexposure among young adults. The predominantly male sample (79.4%) reflects the gender distribution in certain academic programs and may limit generalizability to female students, who may demonstrate different patterns of digital behavior and mental health outcomes [24].

The high academic performance observed in this sample (73.5% with GPAs ≥4.1) suggests that digital wellness challenges may persist even among academically successful students. This underscores the need for institutional strategies that address mental health and digital habits concurrently, rather than assuming that academic success indicates overall well-being.

Implications for practice and policy

These findings have important implications for higher education institutions and public health policy. Universities should consider integrating digital wellness education into curricula and student support services, equipping students to manage their digital behaviors effectively [25]. Institutional support systems should include evidence-based education on healthy technology use, sleep hygiene practices, and stress management techniques, alongside accessible mental health services [26]. From a policy perspective, guidelines promoting healthy digital habits among young adults, particularly those in high-stress academic programs, warrant consideration. Given the strong associations observed, interventions targeting pre-bedtime screen use may be particularly impactful.

Future research directions

Several critical research priorities emerge from these findings. Long-term longitudinal studies are essential to establish temporal relationships and potential causal pathways between digital behaviors and mental health outcomes. Future investigations should incorporate objective measures of screen time, as recorded through device logs, and sleep quality, as measured through actigraphy, to complement self-report data. Randomized controlled trials testing digital wellness interventions, including structured screen time reduction programs and sleep hygiene education, would provide crucial evidence for effective programming. Research examining cultural factors specific to Saudi Arabia and gender differences in digital behavior patterns would further inform targeted intervention development.

Intervention and prevention strategies

Evidence-based intervention strategies warrant implementation across multiple levels. Individual approaches should include digital wellness education programs and sleep hygiene workshops emphasizing pre-bedtime digital restrictions, complemented by mindfulness-based interventions for compulsive social media use. Institutional interventions could encompass university policies that promote digital wellness in dormitories, campus-wide digital detox initiatives, and the integration of digital wellness topics into health education curricula. Policy-level strategies should include national guidelines for healthy digital use, public health campaigns promoting awareness of screen time impacts, and healthcare provider training on digital wellness counseling to create systematic support for addressing these challenges.

Limitations

This study has several important limitations that affect the interpretation of findings. First, the cross-sectional design prevents the establishment of causal relationships between screen time and mental health outcomes; associations observed may reflect bidirectional relationships or confounding variables. Second, convenience sampling rather than true stratified random sampling limits generalizability and may introduce selection bias. Third, the significant gender imbalance (79.4% male) restricts applicability to female university students, who may demonstrate different digital behavior patterns and mental health relationships.

Fourth, reliance on self-reported data for screen time and sleep measures may introduce recall bias and social desirability effects. Objective measures such as device usage logs and actigraphy would provide more accurate assessments. Fifth, the sample was drawn primarily from one university and included students from diverse academic fields beyond health sciences, potentially limiting generalizability to other educational contexts or specifically to health sciences students.

Sixth, no exclusion criteria were applied for pre-existing medical conditions or psychiatric diagnoses, which may confound relationships between digital behaviors and mental health outcomes. Seventh, the sample size (N = 102) was smaller than the calculated requirement (N = 132), potentially reducing statistical power to detect significant associations. Eighth, some chi-square analyses had small expected cell frequencies, violating test assumptions and requiring cautious interpretation of statistical significance.

Finally, validated Arabic versions of mental health instruments were not specifically confirmed, potentially affecting measurement validity in this cultural context. Results should be interpreted with caution, given these methodological limitations.

## Conclusions

This study highlights significant associations between screen time patterns and mental health symptoms among Saudi university students. Specifically, increased social media use and screen exposure before bedtime were linked to higher levels of anxiety, depression, perceived stress, and sleep disturbances. These findings underscore the urgent need to address digital habits as part of student mental health strategies in higher education.

To promote digital wellness, universities should integrate awareness programs into student development initiatives and provide accessible mental health services that include digital behavior counseling. Policymakers and healthcare providers can support these efforts by developing evidence-based guidelines and incorporating digital assessments into routine care. Future research should adopt longitudinal designs and objective measures to further explore and refine interventions aimed at improving student well-being in the context of growing digital engagement.

## Appendices

Introduction to the questionnaire

Study Title: The Impact of Screen Time on Sleep Quality and Mental Health in Saudi University Students

Informed Consent for Participation in Research Study Title: The Impact of Screen Time on Sleep Quality and Mental Health in Saudi University Students Researcher(s): Prof Abdulaziz Al-Kabbaa, Ghaiath Hussein, Mohammed Albader, ⁠Osama Assiri, ⁠Basel Alosaimi3, Muath Al Musaad3, Mohammed Khamsah, Bandar Alzahrani, Abdullah Al Sharani, Nawaf Asiri, Abdulaziz Alotaibi, Abdulrahman Alshahwan Purpose of the Study: This study aims to investigate the relationship between screen time, sleep quality, and mental health among university students in Saudi Arabia. Your participation will help us better understand these relationships and develop strategies to promote healthier digital habits and improve student well-being. What Will Happen During the Study? If you agree to participate, you will be asked to complete an anonymous online questionnaire. The questionnaire will take approximately 10-15 minutes and will include questions about your screen time habits, sleep quality, and mental health. Voluntary Participation: Your participation in this study is completely voluntary. You are free to decline to participate or withdraw from the study at any time without any negative consequences. Your decision to participate or not will not affect your academic standing or any other aspect of your life. Privacy and Confidentiality: All data collected in this study will be anonymous and confidential. Your responses will not be linked to your identity, and no personally identifiable information will be collected. Data will be stored securely on password-protected servers and will only be accessed by the research team. Risks and Benefits: There are no significant risks associated with participating in this study. However, some questions may touch on personal topics such as sleep habits and mental health. If you feel uncomfortable at any time, you may skip questions or withdraw from the study. The benefits of participating include contributing to important research that may help improve the well-being of university students in Saudi Arabia. Debriefing and Support: At the end of the survey, you will receive a debriefing statement with more information about the study and its goals. If you experience any discomfort or need support, you will also be provided with a list of mental health resources available in Saudi Arabia, including contact information for counseling services and helplines. Contact Information: If you have any questions or concerns about the study, please contact the research team at: Email: afkaabba@gmail.com | Phone: +996-0580002284 **Consent Statement:** By clicking "I Agree" below, you confirm that: ·        You have read and understood the information provided above. ·        Your participation is voluntary, and you may withdraw at any time without penalty. ·        You are at least 18 years old. ·        You agree to participate in this study. Debriefing Statement and Mental Health Resources Debriefing Statement: Thank you for participating in this study! Your responses will help us better understand the impact of screen time on sleep quality and mental health among university students in Saudi Arabia. If you have any further questions about the study, please feel free to contact the research team using the contact information provided above. Mental Health Resources in Saudi Arabia: If you are experiencing stress, anxiety, depression, or other mental health concerns, the following resources are available to provide support: 1.     National Mental Health Helpline:   Phone: 920033360 | Available 24/7 for free and confidential support. 2.     Psychological Counseling Services at Your University: Most universities in Saudi Arabia offer free counseling services to students. Contact your university’s student affairs office for more information. 3.     Sehaty App: A government-supported app providing mental health resources, self-help tools, and access to professionals. 4.     Saudi Arabian Association for Mental Health (SAAMH): Website: www.saamh.org.sa | Provides resources, workshops, and support for mental health. 5.     Emergency Mental Health Support: If you are in crisis, visit the nearest hospital or contact emergency services at 911.

Do you agree to participate in the study?

*

Section 1: Demographic Information

What is your age?

What is your sex?

What is your main academic field of study?

What is your year of study?

What is your approximate GPA?

*

After section 2

Section 3 of 5

Section 2: Screen Time Usage

Description (optional)

On average, how many hours per day do you spend on the following devices?

1 or less

2-4

5-6

7-9

10 or more

Smartphone

Laptop/Computer

Tablet

Television

Other screens

What activities do you primarily use screens for? (Select all that apply)

*

Other…

How much time do you spend on social media daily?

How much (average) time do you spend on screens within the hour before bedtime?

After section 3

Section 4 of 5

Section 3: Sleep Quality (Modified Pittsburgh Sleep Quality Index - PSQI)

During the past month, how would you rate your sleep quality overall?

How long (in minutes) does it usually take you to fall asleep each night?

On average, how many hours of actual sleep do you get each night? (Consider interruptions.)

In the past month, how often have you experienced the following:

Not at all

Less than once a week

Once or twice a week

Three or more times a week

Trouble falling asleep within 30 minutes

Waking up in the middle of the night or early morning and having trouble falling back asleep:

Feeling tired or fatigued during the day:

Section 4: Mental Health Indicators

Generalized Anxiety Disorder-7 (GAD-7): Over the last two weeks, how often have you been bothered by the following problems?

0 = Not at all

1 = Several days

2 = More than half the days

3 = Nearly every day

Feeling nervous, anxious, or on edge

Not being able to stop or control worrying

Worrying too much about different things

Trouble relaxing

Feeling restless

Feeling afraid as if something awful might happen

Patient Health Questionnaire-9 (PHQ-9): Over the last two weeks, how often have you been bothered by the following problems?

0 = Not at all

1 = Several days

2 = More than half the days

3 = Nearly every day

Little interest or pleasure in doing things

Feeling down, depressed, or hopeless

Trouble falling or staying asleep, or sleeping too much:

Feeling tired or having little energy

Poor appetite or overeating

Feeling bad about yourself - or that you are a failure or have let yourself or your family down

Trouble concentrating on things, such as reading or watching TV

Moving or speaking so slowly that others noticed, or being so fidgety or restless that you moved around more than usual

Thoughts that you would be better off dead, or of hurting yourself in some way

Perceived Stress Scale (PSS-10): In the last month, how often have you felt or thought the following?

0 = Never

1 = Almost never

2 = Sometimes

3 = Fairly often

4 = Very often

Been upset because of something that happened unexpectedly

Felt unable to control the important things in your life

Felt nervous and "stressed"

Felt confident about your ability to handle personal problems

Felt that things were going your way

Found that you could not cope with all the things you had to do

Been able to control irritations in your life

Felt that you were on top of things

Been angered because of things outside your control

Felt difficulties were piling up so high you could not overcome them

Do you have any additional comments or concerns about your screen time, sleep, or mental health?

Thank You for Your Participation!

Thank You for Your Participation!

Your responses are valuable and will contribute to improving our understanding of the impact of screen time on sleep and mental health among university students.
